# Distinct Binding Dynamics, Sites and Interactions of Fullerene and Fullerenols with Amyloid-β Peptides Revealed by Molecular Dynamics Simulations

**DOI:** 10.3390/ijms20082048

**Published:** 2019-04-25

**Authors:** Zhiwei Liu, Yu Zou, Qingwen Zhang, Peijie Chen, Yu Liu, Zhenyu Qian

**Affiliations:** 1 Key Laboratory of Exercise and Health Sciences (Ministry of Education), School of Kinesiology, Shanghai University of Sport, 399 Changhai Road, Shanghai 200438, China; 15266070790@163.com (Z.L.); chenpeijie@sus.edu.cn (P.C.); yuliu@sus.edu.cn (Y.L.); 2 College of Physical Education and Training, Shanghai University of Sport, 399 Changhai Road, Shanghai 200438, China; zouyu_1993@163.com (Y.Z.); zqw@sus.edu.cn (Q.Z.)

**Keywords:** amyloid protofibril, fullerene, binding site, inhibitory mechanism, molecular dynamics simulation

## Abstract

The pathology Alzheimer’s disease (AD) is associated with the self-assembly of amyloid-β (Aβ) peptides into β-sheet enriched fibrillar aggregates. A promising treatment strategy is focused on the inhibition of amyloid fibrillization of Aβ peptide. Fullerene C_60_ is proved to effectively inhibit Aβ fibrillation while the poor water-solubility restricts its use as a biomedicine agent. In this work, we examined the interaction of fullerene C_60_ and water-soluble fullerenol C_60_(OH)_6_/C_60_(OH)_12_ (C_60_ carrying 6/12 hydroxyl groups) with preformed Aβ_40/42_ protofibrils by multiple molecular dynamics simulations. We found that when binding to the Aβ_42_ protofibril, C_60_, C_60_(OH)_6_ and C_60_(OH)_12_ exhibit distinct binding dynamics, binding sites and peptide interaction. The increased number of hydroxyl groups C_60_ carries leads to slower binding dynamics and weaker binding strength. Binding free energy analysis demonstrates that the C_60_/C_60_(OH)_6_ molecule primarily binds to the C-terminal residues 31–41, whereas C_60_(OH)_12_ favors to bind to N-terminal residues 4–14. The hydrophobic interaction plays a critical role in the interplay between Aβ and all the three nanoparticles, and the π-stacking interaction gets weakened as C_60_ carries more hydroxyls. In addition, the C_60_(OH)_6_ molecule has high affinity to form hydrogen bonds with protein backbones. The binding behaviors of C_60_/C_60_(OH)_6_/C_60_(OH)_12_ to the Aβ_40_ protofibril resemble with those to Aβ_42_. Our work provides a detailed picture of fullerene/fullerenols binding to Aβ protofibril, and is helpful to understand the underlying inhibitory mechanism.

## 1. Introduction

Amyloids are involved in a broad range of neurodegenerative diseases, including Alzheimer’s, Huntington’s and Parkinson’s diseases [1,2,3]. The major constituents of amyloid plaques are associated with fibrils formed by amyloid-β (Aβ) protein that display a cross-β structure characterized by β-strands perpendicular to and inter-strand hydrogen bonds parallel to the fibril axis [4,5]. The fibrillation occurs through a complex multistep process, involving the formation of soluble oligomers, protofibrils and insoluble mature fibrils [6,7]. Small aggregates (soluble oligomers and protofibrils) in the early stage of aggregation are suggested as primary neurotoxic agents [8,9,10,11]. Therefore, a promising strategy to reduce the small toxic oligomer species is to inhibit Aβ peptide aggregation.

The search for effective inhibitors has become an active area of research. An increasing number of experimental and computational studies have reported that Aβ aggregation can be modulated by nanoparticles [12,13,14], small molecules [15,16], short peptides [17,18], antibodies [19] and metal ions [20]. Their findings provided new clues for the design of inhibitors targeting Aβ formation. In recent years, the fullerenes have gained great attention, not only because of their antioxidant, neuroprotective and antitumor properties [21], but also due to their promising ability of carrying contrast agents, radiopharmaceuticals or drugs [22]. However, the poor solubility of fullerenes in water restricts their potential biomedical applications. One of the common strategies to increase of their solubility is to attach hydroxyl groups to the carbon cage, leading to the formation of hydroxylated fullerene (i.e., fullerenol, C_60_(OH)_n_). Fullerenols have high solubility and ability to cross the blood brain barriers [23]. Fullerene derivatives are reported to have remarkable anti-amyloid properties for Alzheimer’s disease and other neurodegenerative diseases [24,25,26,27,28].

Computational studies have investigated the molecular mechanism of fullerenes/fullerenols binding and binding-induced protein remodeling using docking method and molecular dynamics (MD) simulations [29,30,31,32,33]. For example, Li et al. examined the binding affinity of fullerenes in different sizes and found that C_60_ destroys pentameric Aβ_17–42_ fibril structure to a greater extent with respect to other fullerenes [29]. They also found that C_60_(OH)_16_ is inclined to bind at the central hydrophobic and the hydrophobic C-terminal region of monomer Aβ_40_ to prevent amyloid fibrillization [30]. Wei et al. identified three primary binding sites of 1,2-(dimethoxymethano) fullerene (DMF) to Aβ_1–42_ protofibril: the central hydrophobic residues 17–21, the residues 27–31 in turn region and the C-terminal residues 31–41 [31]. They also demonstrated that the fullerene nanoparticles – C_60_ and C_180_ exhibit stronger inhibition on Aβ_16–22_ β-sheet formation [33]. Ding et al. found that different extent of hydroxylation would significantly influence C_60_(OH)_n_–protein interactions [32]. These results reveal the binding modes and inhibitory/disruptive mechanisms of fullerenes/fullerenols, which greatly enhances our understanding of fullerenes/fullerenols-protein interactions at atomic level.

Our previous study examined the influence of DMF on Aβ_42_ dimerization by replica-exchange MD simulations [34]. However, the interactions between Aβ protofibril and fullerenes with different degree of hydroxylation remain elusive. Previous computational study showed that stable Aβ trimer with well-preserved parallel β-strands could act as the smallest seed for Aβ polymerization on self-assembled monolayers [35]. Following the work by Zheng and Wei [35,36], we chose a trimer as Aβ protofibril model in our MD simulations. Here, we investigated the interaction of a C_60_/C_60_(OH)_6_/C_60_(OH)_12_ molecule with Aβ_42/40_ protofibrillar trimer and the resulting protein structural alterations by performing multiple MD simulations. We found that the increased hydroxylation extent of C_60_ leads to slower binding dynamics and weaker binding strength. Binding sites and free energy analyses demonstrate that the C_60_/C_60_(OH)_6_ molecule primarily binds to the C-terminal hydrophobic region, whereas C_60_(OH)_12_ favors to bind to N-terminal residues 4–14. Our simulations revealed the dominant role of hydrophobic interaction in Aβ−nanoparticle interplay. Moreover, the water-soluble C_60_(OH)_6_ molecule has high affinity to form hydrogen bonds with protein backbones, which makes it a more efficient inhibitor than C_60_ and C_60_(OH)_12_.

## 2. Results and Discussion

**Figure 1:**
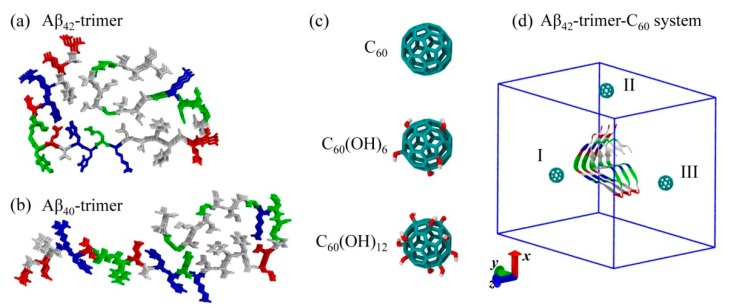
Molecular structures and simulation system setup. (**a**–**c**) The structures of Aβ_42_-trimer, Aβ_40_-trimer and C_60_/C_60_(OH)_6_/C_60_(OH)_12_. (**d**) The initial conformation of the Aβ_42_-trimer-C_60_ system with the C_60_ molecule placed at three different positions (I-III). Color codes: positively charged residues (blue), negatively charged residues (red), hydrophobic residues (white) and polar residues (green) in Aβ peptides; carbon atoms (cyan), oxygen atoms (red) and hydrogen atoms (white) in fullerene/fullerenol. For clarity, water molecules in the simulation box are not displayed; box vectors are shown, and *z*-axis is the fibrillar elongation direction.

We performed MD simulations to study the binding behavior of C_60_/C_60_(OH)_6_/ C_60_(OH)_12_ to Aβ_42_ protofibrillar trimer (Aβ_42_-trimer for short) and Aβ_40_ protofibrillar trimer (Aβ_40_-trimer for short), respectively. The systems are labeled as Aβ_42_-trimer-C_60_, Aβ_42_-trimer-C_60_(OH)_6_, Aβ_42_-trimer-C_60_(OH)_12_, Aβ_40_-trimer-C_60_, Aβ_40_-trimer-C_60_(OH)_6_ and Aβ_40_-trimer-C_60_(OH)_12_. The molecular structures of Aβ_42_-trimer, Aβ_40_-trimer and C_60_/C_60_(OH)_6_/C_60_(OH)_12_ are shown in Figure 1. The initial state of Aβ_42_-trimer-C_60_ system is also displayed, and the other systems are constructed similarly. More details are given in Model and Methods section.

### 2.1. Dynamics of the Fullerene/Fullerenol Molecule Binding to Aβ_42_-Trimer

**Figure 2:**
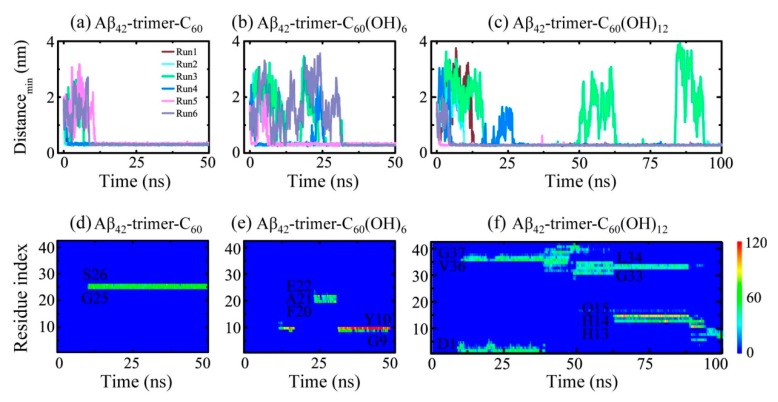
Dynamics of the fullerene/fullerenol molecule binding to Aβ_42_-trimer. (**a**–**c**) Time evolution of the minimum distance between Aβ_42_-trimer and fullerene/fullerenol. Six independent molecular dynamics (MD) runs are denoted in different colors. (**d**–**f**) Time evolution of the number of contacts between individual residue of Aβ_42_-trimer and fullerene/fullerenol in a representative MD run for each simulated system.

To investigate the binding process of the fullerene/fullerenol molecule to Aβ_42_-trimer, we first monitored the time evolution of their minimum distance *d_min_* (Figure 2a–c). As for the Aβ_42_-trimer-C_60_ system, the C_60_ molecule was initially placed 2 nm away from the Aβ_42_-trimer. Once the MD simulations were initiated, *d_min_* started to decrease or increase, depending on the initial velocity distributions. The minimum distances in Run 1, 2 and 4 were observed to decline to ~0.30 nm within the first 3 ns, while those in Run 3, 5 and 6 took ~10 ns to reach ~0.30 nm. Such fast and slow binding processes were also observed in Aβ_42_-trimer-C_60_(OH)_6_ and Aβ_42_-trimer-C_60_(OH)_12_ systems. Similar fast and slow processes were reported in a previous MD study of DMF binding to Aβ fibril [31]. Moreover, we found that the slow binding processes may last tens of nanoseconds for C_60_(OH)_6_ and C_60_(OH)_12_, much longer than that for C_60_. It takes over 25 ns for two MD runs of Aβ-C_60_(OH)_6_ system (Runs 3, 6) to reach a minimum distance of ~0.30 nm, and the situation was the same in Aβ-C_60_(OH)_12_ system (Runs 3, 4). Specially, in Run 3 of Aβ_42_-trimer-C_60_(OH)_12_ system, *d_min_* increased sharply at 49.8 and 83.6 ns, and declined to ~0.30 nm in the next twenty nanoseconds. These indicate that the binding process of the C_60_(OH)_6_/C_60_(OH)_12_ molecule to Aβ_42_-trimer is slower than that of C_60_.

To further examine the binding status of the fullerene/fullerenol molecule after the initial adsorption to Aβ_42_-trimer, we monitored the time evolution of the number of contacts between individual residue and the nanoparticle in a representative MD run for each simulated system in Figure 2d–f. The C_60_ molecule was observed to stay at a relatively fixed location during the remaining simulation time once stable contacts are formed. The C_60_(OH)_6_ molecule also had a relatively fixed binding site, while it can shift to other location transiently. As for the C_60_(OH)_12_ molecule, its binding location kept changing when simulation time increased, corresponding to a slow move on the protein surface. C_60_(OH)_12_ also contacted with more residues at the same time, which indicated a lower specificity of binding sites. These results reflect that with the hydroxylation extent of C_60_ increased, the binding strength between Aβ_42_-trimer and the nanoparticle molecule gets weaker.

In order to quantify the binding strength, we calculated in Table 1 the binding free energy and its different components between Aβ_42_-trimer and the fullerene/fullerenol molecule using the MM/PBSA (molecular mechanics/linear Poisson−Boltzmann surface area) method. The binding energy was calculated over all six MD runs for each simulated system using the last 20 ns data of each MD trajectory. The binding energy components show that the van der Waals interaction (Δ*E_vdW_*) has a dominant contribution to the total binding energy (Δ*G_bind_*). It is shown that Δ*E_vdW_* is -24.02 ± 0.74 kcal/mol in the Aβ-C_60_ system, -24.02 ± 0.74 kcal/mol in the Aβ-C_60_(OH)_6_ system and -18.20 ± 1.02 kcal/mol in the Aβ-C_60_(OH)_12_ system. Interestingly, although C_60_(OH)_6_ carries six more hydroxyl groups than C_60_, their Δ*E_vdW_* is quite similar, and that of C_60_(OH)_12_ became ~6 kcal/mol larger. This reveals that the increment of Δ*E_vdW_* is not in proportion to the hydroxylation level of C_60_ surface. Due to the additional partial charges that hydroxyls bring, the electrostatic interaction (Δ*E*_elec_) is strengthened as the hydroxyl number increases. The nonpolar solvation component Δ*G_nonpolar_* contributes little to the free energy change. The enhanced hydrophilicity with the addition of hydroxyls results in a positive value of Δ*G_solv_* (solvation effect), indicating that water is favorable for fullerenols and solvation effect goes against the binding of fullerenol to Aβ. Our results are consistent with a previous study on fullerenol C_60_(OH)_16_ interacting with Aβ_40_ [30]. They found that the electrostatics contribution is much increased in fullerenol with respect to that in fullerenes, yet hydroxyl groups contribute a positive amount to the binding free energy. Overall, our free energy calculation demonstrates that the total binding free energy rises with more hydroxyl groups attached to C_60_. This gives the explanation that higher hydroxylation level leads to slower binding dynamics and weaker binding strength.

### 2.2. Binding Sites of The Fullerene/Fullerenol Molecule to Aβ_42_-Trimer

**Figure 3:**
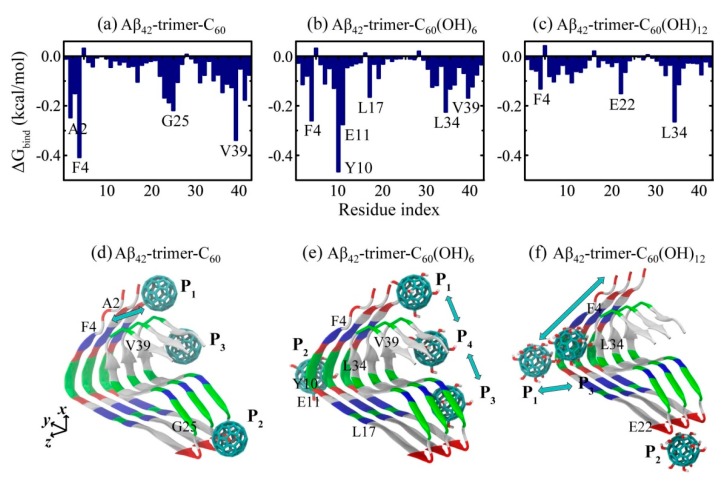
Analysis of binding sites of the fullerene/fullerenol molecule to Aβ_42_-trimer. (**a**–**c**) Residue-based binding free energy. The binding energy was calculated over all six MD runs for each simulated system using the last 20 ns data of each MD trajectory. (**d**–**f**) Schematic diagrams for binding sites of the fullerene/fullerenol molecule to Aβ_42_-trimer. The positions where the fullerene/fullerenol molecule has high binding affinity are named with P_1_, P_2_, etc., from N-termini to C-termini, and *z*-axis is the fibrillar elongation direction. The color code is consistent with that in Figure 1.

Identifying the binding sites of the C_60_/C_60_(OH)_6_/C_60_(OH)_12_ molecule to Aβ_42_-trimer is the first step to understand the underlying inhibition mechanism. To this aim, we calculated the residue-based binding free energy of the nanoparticles to Aβ_42_-trimer in Figure 3a–c using last 20 ns data of the simulations. As shown, C_60_ has the lowest binding energy with aromatic residue F4, and hydrophobic residues V39 and A2, as well as G25 located in the turn region; C_60_(OH)_6_ has the lowest binding energy with aromatic Y10 and F4, negatively charged E11, and hydrophobic L34, L17 and V39; C_60_(OH)_12_ has the lowest binding energy with hydrophobic L34, negatively charged E22 and aromatic F4. This indicates the critical roles of aromatic stacking and hydrophobic interactions in the interplay between Aβ and all the three nanoparticles. As for fullerenols C_60_(OH)_6_ and C_60_(OH)_12_, their hydrogen bonding interaction with negatively charged residues of Aβ is also important.

According to the residue-based binding free energy, we found that C_60_ preferentially interacts with Aβ_42_-trimer at three different sites: 2AEF4, 23DVG25 and C-terminal residues 31–41. Through the binding energy analysis at each site (Table 2), we found that C-terminal residues 31–41 and 2AEF4 have the lowest binding energy, indicating these two regions are the most favorable binding sites for C_60_. This finding is in agreement with the binding sites (aromatic residues F4 and C-terminal hydrophobic residues 31–40) identified in DMF interacting with Aβ dimer [34]. The C-terminal hydrophobic region of residues 31–41 was also reported to be the dominant binding site in DMF interacting with Aβ fibrillar hexamer [31]. As for C_60_(OH)_6_, it prefers to bind to Aβ_42_-trimer at four sites: 2AEF4, 9GYE11, 17LVF19 and C-terminal residues 31–41, among which C-terminal residues 31–41 and 9GYE11 have the lowest binding energy. As for C_60_(OH)_12_, it has three preferential sites: N-terminal residues 4–14, 22ED23 and 34LM35, among which N-terminal residues 4–14 are the most favorable. The hydrophobic clusters A2-F4-L34-V36, L17-F19-I31 and A30-I32-M35-V40 play critical roles in the structural stability of Aβ_42_ fibril [5]. The strong binding of C_60_/C_60_(OH)_6_ to these clusters is expected to interfere with the hydrophobic packing of Aβ side chains, and as a result goes against further fibrillization.

To exhibit the relation between binding dynamics and binding sites clearly, we presented the schematic diagrams for binding sites of C_60_/C_60_(OH)_6_/C_60_(OH)_12_ to Aβ_42_-trimer. The positions where the fullerene/fullerenol molecule has high binding affinity are named with P_1_, P_2_, etc., from *N*-termini to *C*-termini. As shown in Figure 3d, there are three positions P_1_, P_2_ and P_3_ at which C_60_ prefers to stay when binding to Aβ_42_-trimer, mainly corresponding to the binding sties 2AEF4, 23DVG25 and C-terminal residues 31–41, respectively. Note that C_60_ staying at P_1_ can interact with the region 2AEF4 and the *C*-terminal residues 31–41 at the same time. Trajectory tracing shows that C_60_ binds mostly at P_1_ and P_3_ with a respective probability of 22.7% and 29.8%, in agreement with the free energy calculation, and the location of C_60_ is relatively fixed. Moreover, the C_60_ molecule is able to wander on the surface groove along *z*-axis of Aβ_42_-trimer at P_1_ position. These preferential positions are near two hydrophobic clusters A2-F4-L34-V36 and A30-I32-M35-V40, indicating that the binding of C_60_ to Aβ_42_-trimer is dominantly driven by the hydrophobic interaction. The importance of hydrophobic interaction was reported in other studies on the binding processes of fullerene and other small molecules to Aβ [29,37,38]. With respect to P_1_ and P_3_, C_60_ has a relatively lower binding affinity to P_2_. This binding site is facilitated by the groove of a proper size in the 23DVGS26 region, where the side chains of D23 and S26 are in the outer side of Aβ_42_-trimer and G25 has no side chain. Similar concave-induced binding sites were observed in the study of fullerenes with Aβ and other proteins [31,39,40].

As for C_60_(OH)_6_, it has four preferential binding positions P_1_, P_2_, P_3_ and P_4_ (Figure 3e), mainly corresponding to the binding sties 2AEF4, 9GYE11, 17LVF19 and C-terminal residues 31–41, respectively. Interestingly, the C_60_(OH)_6_ molecule is able to slip on the elongation surface (perpendicular to *z*-axis), wandering between P_1_ and P_4_ or between P_3_ and P_4_ with a low probability. The P_3_ position is adjacent to another hydrophobic cluster L17-F19-I31 of Aβ_42_-trimer. Besides, C_60_(OH)_6_ has high binding affinity to 9GYE11 (P_2_), facilitated by the hydrogen bonds (H-bonds) formed in between. As for C_60_(OH)_12_, it prefers to bind to three positions P_1_, P_2_ and P_3_ (Figure 3f), corresponding to the binding sties N-terminal residues 4–14, 22ED23 and 34LM35, respectively. Different from the binding behaviors of C_60_ and C_60_(OH)_6_, C_60_(OH)_12_ is more likely to stay at the hydrophilic parts of protein surface. It is able to move between positions P_1_ and P_3_, or slip along the N-terminal β-strand, forming H-bonds with main chain or side chain of amino acids. The C_60_(OH)_12_ molecule may also contact with 22ED23 region. As the side chains of E22 and D23 are oriented to water solution, C_60_(OH)_12_ is inclined to form H-bonds with them. Considering the important roles of C-terminal hydrophobic residues in Aβ aggregation and toxicity [41,42,43], it is conceivable that the binding of C_60_ and C_60_(OH)_6_ molecules to the C-terminal region can prevent Aβ fibrillization. In addition, the C_60_(OH)_6_ molecule has higher affinity to bind to elongation surfaces than C_60_ and C_60_(OH)_12_, which makes C_60_(OH)_6_ a more effective inhibitor. As previous computational and experimental studies suggested that binding at fibril ends goes against fibrillar elongation [44,45,46], this binding would block the backbone amide sites for fibril growth and as a result, slows down or inhibits the elongation process. It is noted that the bindings of nanoparticles to protofibril and mature fibril are supposed to be distinct, because the relative area of the exposed ends compared to the entire fibril surface will be greatly decreased in mature fibrils.

### 2.3. Structural Influence of The Fullerene/Fullerenol Molecule on Aβ_42_-Trimer

**Figure 4:**
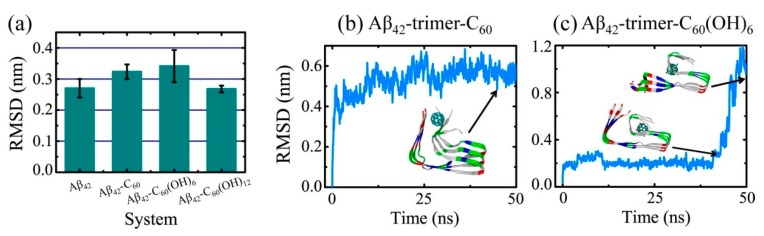
Influence of fullerene/fullerenol molecules on the Aβ_42_-trimer structure. (**a**) The average Cα-root-mean-square deviation (Cα-RMSD) relative to the initial coordinates of Aβ_42_ protofibrillar trimer. The values for Aβ-fullerene/fullerenol systems were calculated over all six MD runs for each simulated system using the last 20 ns data of each MD trajectory, and those for isolated Aβ_42_-trimer systems were averaged over the last 50 ns data of two independent 200-ns MD runs. (**b**,**c**) Time evolution of Cα-RMSD of the MD trajectory that contributes most to the total Cα-RMSD in Aβ_42_-trimer-C_60_ and Aβ_42_-trimer-C_60_(OH)_6_ systems, respectively. The color code of the inset snapshots is consistent with that in Figure 1.

In order to detect the influence of fullerene/fullerenol binding on the Aβ_42_-trimer structure, we first examined the secondary structural difference relative to the isolated Aβ_42_-trimer. The β-sheet contents of Aβ_42_-trimer, Aβ_42_-trimer-C_60_, Aβ_42_-trimer-C_60_(OH)_6_ and Aβ_42_-trimer-C_60_(OH)_12_ systems are 80.5%, 83.3%, 81.1% and 80.4%, respectively, showing little difference. Then, we calculated the average Cα-root-mean-square deviation (Cα-RMSD) with respect to the initial coordinates of Aβ_42_ protofibrillar trimer using the last 20 ns data of each MD trajectory. As shown in Figure 4a, the values of Cα-RMSD in the absence and presence of the C_60_(OH)_12_ molecule are 0.27 ± 0.03 nm and 0.27 ± 0.01 nm, showing no statistically significant difference. In the presence of C_60_/C_60_(OH)_6_, Aβ_42_-trimer has an increased Cα-RMSD of 0.32 ± 0.02 / 0.34 ± 0.05 nm, while the values are still within the error of estimate with respect to that of isolated Aβ. These indicate that the C_60_/C_60_(OH)_6_/C_60_(OH)_12_ molecule has a negligible influence on the structural stability of Aβ_42_-trimer.

Figure 4b,c display the time evolution of Cα-RMSD of the MD trajectory contributing most to the total Cα-RMSD in Aβ_42_-trimer-C_60_ and Aβ_42_-trimer-C_60_(OH)_6_ systems, respectively. With C_60_, the Cα-RMSD value of Aβ_42_-trimer keeps rising in the first 20 ns and finally fluctuates at around 0.55 nm. During this process, the C_60_ molecule is observed to contact abundantly with side chains of V39 and I41, and lead to twisted C-termini. In the Aβ_42_-trimer-C_60_(OH)_6_ system, the Cα-RMSD value of Aβ_42_-trimer keeps at ~0.30 nm until *t* = 40.8 ns. After that, it rises sharply and increases to >1.0 nm. When Cα-RMSD begins its quick rise, the C_60_(OH)_6_ molecule is observed to bind at the C-terminal residues 31–41, and the hydrophobic cluster A2-F4-L34-V36 starts to collapse. Then, the sidechains of A2 and F4 dissociate with those of L34 and V36 one by one, and finally the N-termini and C-termini get separated far away. Note that it is the only MD trajectory among all the simulations we performed in this study that N- and C-termini dissociation is observed. It needs further studying to connect this dissociation with Aβ-C_60_(OH)_6_ interaction explicitly.

**Figure 5:**
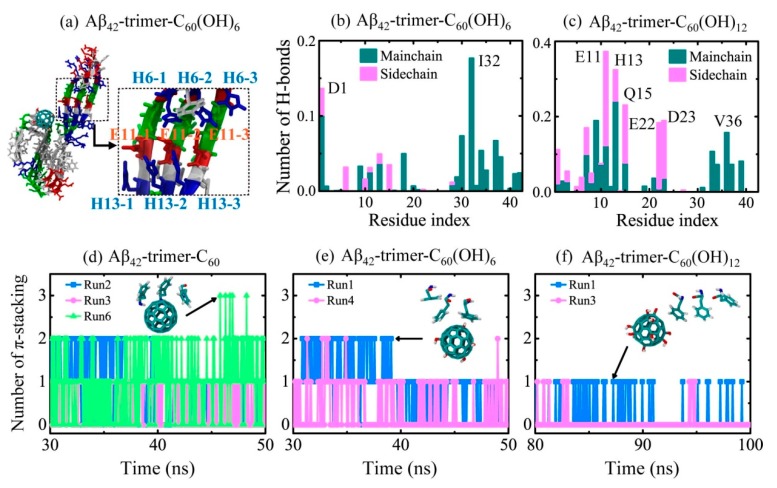
Details of interactions between Aβ_42_-trimer and the fullerene/fullerenol molecule. (**a**) Disturbance to the interplay of H6-E11-H13 observed in a trajectory of Aβ_42_-trimer-C_60_(OH)_6_ system. (**b**,**c**) Number of H-bonds formed between Aβ_42_-trimer and fullerenols. (**d**–**f**) Number of π-stacking structures between Aβ_42_-trimer and fullerene/fullerenol. The geometrical criterions of H-bonding and π-stacking formations are defined in Model and Methods section.

The detailed interactions between Aβ_42_-trimer and the fullerene/fullerenol molecule were also investigated. As a previous study suggested that the salt bridges between H6, E11 and H13 stabilize the kink in the N-terminal part of the β-sheets around Y10 [5], we examined the interplay of H6-E11-H13 and found that the interaction pairs stably stay together in all simulated systems except for one MD trajectory of Aβ_42_-trimer-C_60_(OH)_6_ system. This trajectory corresponds to the MD run shown in Figure 4c, and its snapshot of the final state is presented in Figure 5a. Even if the *N*- and *C*-termini are dissociated, the interaction pairs of H6, E11 and H13 mostly stay together, and the interactions of side chains are weakened by excluding those of H6-3 (H6 in Chain 3) and E13-1 (E13 in Chain 1).

In Figure 5b,c, we calculated the number of H-bonds formed between individual residue and fullerenols. It shows that C_60_(OH)_6_ favors H-bonding with main chains of Aβ_42_-trimer, and forms H-bonds mostly with residues I32 and D1. The C_60_(OH)_12_ molecule forms almost the same amount of H-bonds with main chains and side chains, and it preferentially forms H-bonds with residues E11, H13, Q15, D23, E22 and V36. Previous Thioflavin T (ThT) fluorescence and atomic force microscopy experiments showed that fullerenol C_60_(OH)_16_ can prevent Aβ_40_ fbrillization [30]. The recent study using ThT assay and transmission electron microscope demonstrated that fullerenemalonate can inhibit Aβ_42_ aggregation [47]. Their computational results showed that the inhibition is attributed to the hydrogen bonding of the fullerenemalonate carboxylate groups with Aβ. Here, the formation of H-bonds between main chains and fullerenols is supposed to block the backbone amide sites for further addition of peptides in β-sheet structure, which goes against the oligomerization or fibrillization of Aβ. The higher affinity of C_60_(OH)_6_ bonding with main chains of Aβ peptides makes C_60_(OH)_6_ a more efficient inhibitor than C_60_(OH)_12_.

The π-stacking interaction is important in the self-assembly of amyloid fibrils, with parallel, T-shaped and herringbone (~50°) orientations suggested for aromatic rings in proteins [48]. The binding energy analysis reveals the important role of F4 in the interaction between Aβ_42_-trimer and the fullerene/fullerenol molecule. To examine the aromatic stacking interaction between F4 and C_60_/C_60_(OH)_6_/C_60_(OH)_12_, we calculated the number of π-stacking structures between Aβ42-trimer and fullerene/fullerenol during the last 20 ns in Figure 5d–f. For the Aβ_42_-trimer-C_60_ system, π-stacking structures were observed in three MD trajectories. Run 6 had the largest number of π-stacking structures, and the maximum number was three. This means that the C_60_ molecule is able to have π-stacking interaction with all the aromatic rings of F4 in Aβ_42_-trimer at the same time. The inset snapshot displays the corresponding structure, and the aromatic rings of F4 are oriented in parallel or herringbone alignment relative to the C_60_ surface. As for C_60_(OH)_6_, it forms less π-stacking structures with Aβ_42_-trimer, and the maximum number of π-stacking decreases to two. For the Aβ_42_-trimer-C_60_(OH)_12_ system, π-stacking structures are observed in two trajectories and the total number of π-stacking structures is the least. Only one aromatic ring of F4 can have π-stacking interaction with the C_60_(OH)_12_ molecule at one moment, and the ring is mostly oriented in herringbone alignment relative to the carbon surface of C_60_(OH)_12_. These results indicate that the more hydroxylated C_60_ is, the fewer and weaker π-stacking interactions with Aβ_42_-trimer the nanoparticle has.

### 2.4. Dynamics, Sites and Interactions of The Fullerene/Fullerenol Molecule Binding to Aβ_40_-Trimer

**Figure 6:**
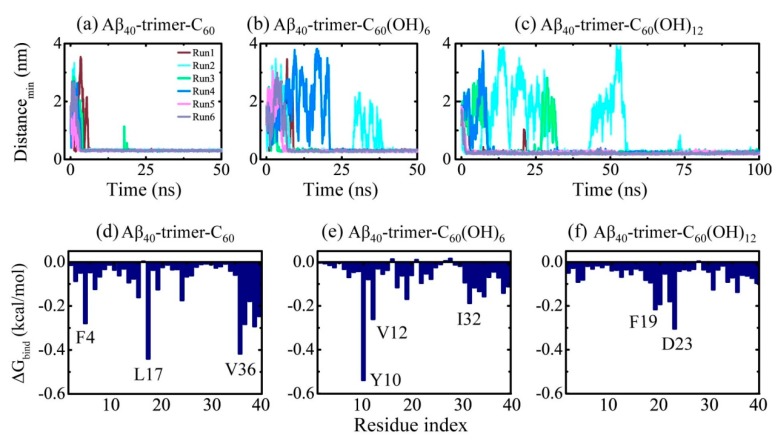
(**a**–**c**) Time evolution of the minimum distance between Aβ_40_-trimer and fullerene/fullerenol. Six independent MD runs are denoted in different colors. (**d**–**f**) Residue-based binding free energy. The binding energy was calculated over all six MD runs for each simulated system using the last 20 ns data of each MD trajectory.

We also carried out multiple MD simulations to examine the binding dynamics, binding sites and interactions of the C_60_/C_60_(OH)_6_/C_60_(OH)_12_ molecule with Aβ_40_-trimer. Although the structure of Aβ_40_-trimer is different from that of Aβ_42_-trimer (see Figure 1), the binding behavior of nanoparticles to Aβ_40_-trimer was found to display a remarkable resemblance with that to Aβ_42_-trimer. As shown in Figure 6, with the hydroxylation extent increased, the C_60_ molecule displays slower binding dynamics, corresponding to weakened binding strength. The binding free energy analysis shows that the favorable residues of Aβ_40_-trimer with which the nanoparticle tend to interact are a little different from those of Aβ_42_-trimer. Still, these residues are mostly hydrophobic or aromatic, indicating the critical roles of hydrophobic and aromatic interactions in Aβ-nanoparticle interactions. Moreover, the preferential binding regions of the nanoparticles interplaying with Aβ_40_-trimer resemble with those of the nanoparticles binding to Aβ_42_-trimer. We also examined the stability of the D23-K28 salt bridge, which is important for the structural stability of Aβ_40_ [4]. The salt bridge would be interfered by the nanoparticle binding, whereas the connection between the salt bridge disruption and the hydroxylation extent of C_60_ is not explicit.

## 3. Materials and Methods

### 3.1. Aβ_40/42_ Protofibrillar Trimer and C_60_/C_60_(OH)_6_ /C_60_(OH)_12_ Molecules

The Aβ peptide (39–43-amino acid) is derived from the amyloid precursor protein (APP) through proteolytic cleavage by β- and γ-secretase, and the most abundant Aβ are Aβ_42_ (sequence: DAEFRHDSGY^10^EVHHQKLVFF^20^AEDVGSNKGA^30^IIGLMVGGVV^40^IA) and Aβ_40_. The initial coordinate of the Aβ_42_ protofibrillar trimer was taken from the Aβ_42_ fibril structure [5] (PDB ID: 5OQV) determined by cryo–electron microscopy (cryo-EM). The coordinate of the Aβ_40_ protofibrillar trimer was taken from the Aβ_40_ fibril structure [4] (PDB ID: 2M4J) obtained from solid-state nuclear magnetic resonance (NMR) spectroscopic data. The protonation of the peptide was adjusted to the neutral pH. The *N*- and *C*-termini were respectively capped by NH_3_^+^ and COO^−^ in accordance with experiments.

The structure of C_60_/C_60_(OH)_6_/C_60_(OH)_12_ molecules used in this study is displayed in Figure 1. The force field parameters were taken from a previous MD study on the interaction of Aβ and hydroxylated carbon nanotube [49]. To simplify the modeling, the hydroxyl groups in C_60_(OH)_6_/C_60_(OH)_12_ molecules are distributed uniformly on the C_60_ surface.

The Aβ_42_-trimer-C_60_ simulation system consists of an Aβ_42_ protofibrillar trimer and a C_60_ molecule placed 2.0 nm (minimum distance) away from Aβ, as shown in Figure 1. To remove the bias of the initial position of C_60_ on the binding site, the C_60_ molecule was initially placed at three different locations (I, II, III). The other Aβ-fullerene/fullerenol systems were constructed similarly, and were immersed in SPC [50] water. Counterions Na^+^ and Cl^-^ were added to neutralize the system and provide an additional 0.1 M salt concentration. Systems of isolated Aβ_42_-trimer and Aβ_40_-trimer in water were run as control groups.

### 3.2. Details of MD Simulations

Atomistic MD simulations were performed in isothermal−isobaric (NPT) ensemble using GROMACS-4.5.3 software package [51] with GROMOS96 53a6 force field [52], in accordance with previous computational studies of Aβ peptides [31,33,34,49,53,54]. Periodic boundary conditions were applied in all three directions. The temperature and pressure of the systems were coupled using the Nose−Hoover algorithm [55,56] (310 K, τ_T_ = 0.2 ps) and Parinello–Rahman algorithm [57,58] (1 bar, τ_P_ = 1.0 ps), respectively. The simulation time step was 2 fs with all bonds constrained using the LINCS algorithm [59]. The electrostatic interactions were treated with the particle mesh Ewald (PME) method [60] with a cutoff of 1.0 nm, and the van der Waals interactions were calculated using a cutoff of 1.4 nm. For Aβ_40/42_-trimer-C_60_ and Aβ_40/42_-trimer-C_60_(OH)_6_ systems, six independent copies of each system were carried out, each lasting 50 ns; for Aβ_40/42_-trimer-C_60_(OH)_12_ systems, six independent 100-ns MD runs were carried out; for isolated Aβ_40/42_-trimer systems, two independent 200-ns MD runs were carried out.

### 3.3. Analysis Methods

Trajectory analysis was performed using the GROMACS-4.5.3 package toolkits and in-house developed codes. The secondary structure was calculated using the DSSP program [61]. Here, an atomic contact is defined when two non-hydrogen atoms come within 0.54 nm. The H-bond is determined using geometrical criteria: the distance between donor D and acceptor A is less than 0.35 nm and the D-H-A angle is larger than 150°. The π-stacking structure is defined when the centroid of residue aromatic ring is within 0.45 nm from the spherical carbon surface of fullerene/fullerenol [62]. The binding energy between a ligand and a receptor was estimated by means of (MM/PBSA) [63,64]: Δ*G_bind_* = Δ*E_MM_* + ΔG_solv_ − TΔS, Δ*E_MM_* = Δ*E_vdW_* + Δ*E_elec_*, Δ*G_solv_* = Δ*G_polar_* + Δ*G_nonpolar_*, Δ*G_nonpolar_* = γ·SASA + b. Here, *E_MM_* is the gas-phase energy, consisting of electrostatic (Δ*E*_elec_) and van der Waals (Δ*E*_vdw_) terms; Δ*G_solv_* is the sum of polar solvation energy Δ*G_polar_* and nonpolar solvation component Δ*G_nonpolar_*; Δ*G_polar_* is estimated by solving the Poisson−Boltzmann equation; Δ*G_nonpolar_* is estimated by solvent accessible surface area (SASA). A water probe radius of 0.14 nm was used to calculate SASA, and γ (surface tension of the solvent) and b (fitting parameter) were set to 0.542 kcal/mol/nm^2^ and 0.92 kcal/mol, respectively. As the binding free energy (Δ*G_bind_*) reported here is the relative binding free energy, the contribution of conformational entropy of peptides was ignored in accordance with a number of previous computational studies [33,34,65,66].

## 4. Conclusions

We investigated the dynamics, sites and interactions of the C_60_/C_60_(OH)_6_/C_60_(OH)_12_ nanoparticle binding to Aβ_42/40_ protofibrillar trimer by performing extensive atomistic MD simulations. To our knowledge, this is the first atomistic explicit-solvent simulation study to investigate the binding behavior of fullerenols to Aβ_42/40_ protofibril. Our simulations demonstrate that the higher hydroxylation level of C_60_ leads to slower binding dynamics and weaker binding strength. When binding to Aβ_42_-trimer, C_60_ preferentially interacts with C-terminal residues 31–41 and 2AEF4; C_60_(OH)_6_ prefers to bind to C-terminal residues 31–41 and 9GYE11; C_60_(OH)_12_ favors to bind to *N*-terminal residues 4–14. In addition, the C_60_(OH)_6_ molecule has higher affinity to bind to elongation surfaces than C_60_ and C_60_(OH)_12_. The binding of these nanoparticles has a slight influence on the secondary structure and structural stability of Aβ_42_-trimer during the simulation time. The hydrophobic interaction plays a critical role in the interplay between Aβ_42_ and all three nanoparticles; π-stacking interaction gets weakened as C_60_ carries more hydroxyls. The situations are quite similar when the C_60_/C_60_(OH)_6_/C_60_(OH)_12_ nanoparticle binds to Aβ_40_ protofibrillar trimer. Overall, the proper binding strength and high affinity to form hydrogen bonds with protein backbones make the water-soluble C_60_(OH)_6_ molecule an efficient inhibitor. This study provides a detailed picture of fullerene/fullerenols binding to Aβ protofibril and expands the understanding of the underlying inhibitory mechanism, which is helpful to the design of novel agents with anti-amyloid properties.

## Figures and Tables

**Table 1:** Different components of binding free energy (in kcal/mol) between Aβ_42_-trimer and the fullerene/fullerenol molecule.

Systems	ΔE_vdw_	ΔE_elec_	ΔE_MM_	ΔG_polar_	ΔG_nonpolar_	ΔG_solv_	ΔG_bind_
Aβ_42_-trimer-C_60_	−24.44 ± 0.69	0	−24.44 ± 0.69	0	−3.92 ± 0.16	−3.92 ± 0.16	−28.36 ± 0.71
Aβ_42_-trimer-C_60_(OH)_6_	−24.02 ± 0.74	−5.16 ± 0.69	−29.18 ± 0.25	15.27 ± 1.68	−3.61 ± 0.16	11.66 ± 1.69	−17.52 ± 1.71
Aβ_42_-trimer-C_60_(OH)_12_	−18.20 ± 1.02	−14.60 ± 1.45	−32.80 ± 1.77	27.06 ± 2.52	−3.30 ± 0.17	23.77 ± 2.53	−9.03 ± 3.09

**Table 2:** Free energy (in kcal/mol) of different binding sites for the fullerene/fullerenol molecule to Aβ_42_-trimer.

System	Aβ_42_-trimer-C_60_			Aβ_42_-trimer-C_60_(OH)_6_				Aβ_42_-trimer-C_60_(OH)_12_		
Binding site	2–4	23–25	31–41	2–4	9–11	17–19	31–41	4–14	22–23	34–35
ΔG_bind_	−0.80	−0.57	−1.32	−0.45	−0.87	−0.28	−1.23	−0.73	−0.21	−0.38
Deviation	0.09	0.02	0.01	0.06	0.03	0.04	0.05	0.07	0.04	0.05
